# Changes in inpatients' perceptions of the quality of smart healthcare services at county-level public hospitals in Chongqing: a repeated cross-sectional study, 2019-2025

**DOI:** 10.3389/fpubh.2026.1942631

**Published:** 2026-10-21

**Authors:** Siyu Wen, Wei Gu, Quanxin Deng, Binyuan Ma, Jing Li

**Affiliations:** Center for Medical and Social Development Research, School of Public Health, Chongqing Medical University, Chongqing, China

**Keywords:** county-level public hospitals, service quality, SERVQUAL model, smart healthcare, temporal trends

## Abstract

**Objective:**

This study examined temporal changes in the quality of smart healthcare services perceived by inpatients at county-level public hospitals in Chongqing across three survey waves conducted in 2019, 2021, and 2025, and assessed differences across patient subgroups. The findings may inform evaluations of smart healthcare development and the ongoing optimization of healthcare services.

**Methods:**

Questionnaire data collected from inpatients at six county-level public hospitals were analyzed. A SERVQUAL-based instrument assessed patients' expectations and perceptions across reliability, assurance, responsiveness, economic, empathy, and tangibles, and calculated service quality scores accordingly. Analysis of variance was used to assess trends in each dimension, while multivariable linear regression and interaction analyses were used to examine temporal changes in service quality scores and differences across subgroups.

**Results:**

A total of 855 inpatients participated in this study. The mean gap between patients' perceived and expected service quality increased from −0.72 ± 0.60 in 2019 to −0.46 ± 0.47 in 2025, differences among the three survey waves were statistically significant (*P* < 0.001). Among the six dimensions, assurance (−0.76 to −0.39), reliability (−0.73 to −0.41), tangibles (−0.64 to −0.34), responsiveness (−0.63 to −0.40), and empathy (−0.65 to −0.42) all showed a significant improvement (*P* < 0.001). The economic dimension showed the smallest numerical change (−0.93 to −0.82), differences across the three survey waves were not statistically significant (*F* = 1.390, *P* = 0.250). Service quality scores differed significantly by survey year, age, educational attainment, monthly income, and residence. In particular, patients aged 60 years or older (*B* = −0.212, *P* < 0.001) and those with a bachelor's degree or higher (*B* = −0.117, *P* = 0.019) reported lower scores.

**Conclusion:**

Perceived smart healthcare service quality improved across the three survey waves in county-level public hospitals in Chongqing, and the gap between patients' expectations and perceptions gradually narrowed. However, improvement in the economic dimension was limited. These findings underscore the importance of continuing to advance technological upgrades while promoting the mutual recognition of diagnostic test results across institutions, exploring communication tools such as intelligent consultation systems and bedside interactive terminals, and delivering smart healthcare services tailored to diverse patient needs.

## 1 Introduction

Smart healthcare refers to the use of advanced information technologies, such as artificial intelligence, big data, cloud computing, and the Internet of Things, to modernize healthcare services, improve service efficiency, enhance patient experience, and increase access to healthcare (1, 2). In recent years, the Chinese government has actively promoted the development of smart healthcare through a series of national policies. In the “Action Plan for Promoting High-Quality Development of Public Hospitals (2021–2025),” the National Health Commission explicitly called for promoting the integrated development of electronic medical records, smart services, and smart management to improve hospital service quality and public satisfaction (3). Guided by national policies, the Chongqing Municipal Health Commission proposed in the “14th Five-Year Plan for Health Informationization Development in Chongqing” that by 2025, a nationally leading health information infrastructure system would be established, positioning Chongqing as a nationally recognized model for smart healthcare (4).

Service quality is central to patient-centered healthcare, and the SERVQUAL model is currently one of the most widely used tools for assessing service quality in healthcare (5, 6). Proposed by Parasuraman et al. (7) this model evaluates service quality by comparing the gap between patient expectations and actual perceptions, covering five dimensions: tangibles, reliability, responsiveness, assurance, and empathy. As the model was originally developed in a western commercial service context, its dimensional structure may not fully adapt to healthcare environments shaped by different institutional arrangements, and applying it in China therefore requires adjustment based on the country's national conditions and the specific characteristics of its healthcare industry. Reflecting this need, previous studies have drawn on the perceived service quality model (8), based on which Niu Hongli argued that patients evaluate not only the process of care delivery but also its outcome, including their psychological tolerance of the price of the corresponding service. Accordingly, when applying the SERVQUAL model to China's healthcare system, these studies adapted and expanded it by adding an economic dimension to the traditional five dimensions, incorporating cost-related factors, including medical expenses, into service quality assessment (9, 10). This is particularly relevant in China: although out-of-pocket expenditure as a share of total health spending has declined, it still accounts for a substantial proportion of household spending (11), constituting a considerable economic burden for patients. In addition, the development of smart healthcare has transformed the way medical services are delivered, prompting patients to place greater emphasis on the reasonableness of medical costs and the value of the care they receive. Therefore, drawing on existing research, this study adds the “economic” dimension to the traditional five-dimensional SERVQUAL model to more comprehensively evaluate patients' service experiences in the context of smart healthcare.

As the only municipality directly under the central government in western China, Chongqing launched a pilot program for smart hospitals in 2018 to assess the actual effectiveness of smart healthcare development across the city's public hospitals. Following this initiative, the number of smart hospitals increased. However, hospital digitalization remains uneven across regions, and county-level public hospitals have relatively limited information technology infrastructure and digital capacity (12, 13). Chongqing encompasses a large metropolitan area, extensive rural and mountainous areas, and a large reservoir region, and has a pronounced urban-rural divide. Of its 34 million registered residents, nearly 17 million are registered rural residents. County-level public hospitals serve as key hubs in China's tiered healthcare system and provide essential healthcare services for residents of rural and peri-urban areas. Therefore, evaluating the quality of smart healthcare services at this level holds significant practical importance.

Previous studies have largely focused on the technological implementation of smart healthcare (2) or conducted cross-sectional surveys at a single point in time to evaluate patient service experiences (14). However, evidence on temporal changes in patients' perceived service quality remains limited, as does evidence on whether these changes differ across demographic groups. Therefore, this study used a repeated cross-sectional design to examine county-level public hospitals in Chongqing. The study included three survey waves, conducted in 2019, 2021, and 2025, representing distinct stages of smart healthcare development. To compare changes in patients' perceived service quality across the three survey waves and examine differences in ratings across demographic subgroups. The study examined whether changes in perceived service quality over time coincided with the ongoing development of smart healthcare. The findings may also inform smart healthcare development and efforts to improve patient experience in other resource-limited settings.

## 2 Methods

### 2.1 Study design

This study used a repeated cross-sectional design to evaluate temporal trends in perceived smart healthcare service quality among inpatients at county-level public hospitals in Chongqing. Three cross-sectional survey waves were conducted in 2019, 2021, and 2025. The same eligibility criteria, sampling strategy, and survey instrument were used across all three survey waves to ensure comparability across years.

The three survey years were selected to reflect the policy implementation process and distinct stages of smart healthcare development in Chongqing. In 2018, Chongqing launched the “Smart Hospital Demonstration Project (Pilot Phase).” The 2019 survey represented the early implementation stage and was used to assess patients' perceptions of service quality at the outset of smart healthcare development. In 2021, the “Action Plan for Promoting High-Quality Development of Public Hospitals (2021–2025)” and the “14th Five-Year Plan for Health Informatization Development in Chongqing” were successively implemented, driving the integrated development of electronic medical records, smart services, and smart management. The 2021 survey represented a phase of accelerated smart healthcare implementation. The 2025 survey served as a key evaluation point in the implementation cycle of these plans, used to observe changes in patients' perceptions of service quality during the ongoing policy implementation period.

### 2.2 Sample size calculation

According to Kendall's sample size estimation method (15), the required sample size should be 10 times the number of items included in the analysis. The questionnaire used in this study comprised 24 items. To account for sampling error and invalid questionnaires, the sample size was further expanded by 20%, yielding a minimum required sample size of 288. Accordingly, a target sample size of 300 participants per survey wave was adopted.

### 2.3 Data collection

From October to November 2019, based on the level of smart hospital development and local economic development across Chongqing's districts and counties, this study employed a stratified random sampling method to conduct a survey at six county-level public hospitals located in the southwestern, northern, and southeastern parts of Chongqing that had been rated as smart hospitals by the municipal government. These hospitals had already established core smart healthcare infrastructure and service systems prior to the first survey wave, and the core smart healthcare services assessed in the questionnaire remained relatively consistent across all hospitals and waves. In each survey wave, fifty inpatients were randomly selected from 5 ~ 6 departments in each hospital, and trained interviewers administered the questionnaires face-to-face. Questionnaire administration took place during a clinically stable phase of the patient's hospitalization. From October to November 2021 and 2025, the study continued to use the same hospitals and sampling framework established in 2019; that is, all three rounds of the survey were conducted at the same six hospitals to ensure consistency in the source of the study participants. Interviewers received standardized professional training from the same supervising researcher prior to each wave to ensure familiarity with the questionnaire content. Questionnaires were distributed after obtaining participants' consent and completed independently by the participants. If participants had questions about items, interviewers explained the meaning of the items to ensure correct understanding, without expressing or implying personal opinions, so as to avoid information bias. Inclusion criteria for study participants were: (1) having experienced smart healthcare-related services during hospitalization (e.g., self-service devices or the hospital's mobile platform for record inquiry, payment, or online doctor-patient communication), confirmed via a screening question; (2) having adequate cognitive function and the ability to communicate verbally; (3) being willing to participate in the study. Exclusion criteria included: patients who were too ill to complete the survey and those with altered consciousness or communication impairment.

### 2.4 Questionnaire design

To ensure comparability across survey waves, this study used a questionnaire previously developed and finalized by the research team for a related project, “Development of a SERVQUAL-Based Service Quality Evaluation Indicator System for County-Level Public Hospitals in Chongqing” (16). The questionnaire was based on the SERVQUAL model and informed by the Chongqing Smart Hospital Demonstration Construction Evaluation Indicators (17) and expert consultation, and was revised and refined following a pilot survey. The overall Cronbach's α coefficient for the questionnaire was 0.970, and the content validity index (S-CVI/Ave) was 0.980, indicating good internal consistency and content validity.

The questionnaire comprised two sections: (1) a general information section covering gender, age, educational level, occupation, monthly income, health insurance type, and place of residence; (2) a smart healthcare service quality questionnaire, which assesses patients' expectations and perceptions of smart healthcare services across six dimensions—reliability, assurance, responsiveness, economic, empathy, and tangibles—comprising a total of 24 items. These items covered the smart healthcare services and infrastructure established at the study hospitals, including self-service terminals and smart devices; hospital mobile platforms for fee inquiries, examination reminders, and access to inpatient and test records; internet hospital and online doctor-patient communication channels; intelligent diagnosis and treatment support systems; online payment platforms; and mechanisms for patient feedback and privacy protection. A five-point Likert scale was used, with scores ranging from one (very low expectation or very dissatisfied) to five (very high expectation or very satisfied).

In the study sample, the Cronbach's α coefficients for the Expectations Questionnaire and the Perceptions Questionnaire were 0.934 and 0.959, respectively, indicating good internal consistency. The Kaiser-Meyer-Olkin values for the expectation and perception scales were 0.930 and 0.958, respectively. Bartlett's test of sphericity was statistically significant for both scales (*P* < 0.001). The cumulative proportions of variance explained were 61.2% and 64.2%, respectively. Together, these findings support the reliability and construct validity of the scales.

### 2.5 Calculation of Smart Healthcare Service Quality (SQ) Scores

$$\begin{array}{l} {\text{SQ}}_{j}=\frac{1}{k}\overset{k}{\underset{i=1}{\sum }}\left(P_{ji}-E_{ji}\right) \end{array}$$

For each participant *j*, the SQ score was calculated as the mean difference between perceived and expected scores across the relevant items (Equation 1), where *k* denotes the number of items (*k* = 24 for the overall SQ score, or *k* = 4 for each of the six dimension scores) and *P*_ji_ and *E*_ji_ are the perceived and expected scores of participant *j* on item *i*. Higher SQ scores indicate a smaller gap between perceived and expected service quality and, consequently, better perceived smart healthcare service quality. The specific calculations for each dimension and the overall score are shown in Table 1.

**Table 1:** Calculation method for the overall and dimension SQ scores.

Dimensions	Calculating SQ scores
Reliability	=[(P1-E1) + (P2-E2) + (P3-E3) + (P4-E4)]/4
Assurance	=[(P5-E5) + (P6-E6) + (P7-E7) + (P8-E8)]/4
Responsiveness	=[(P9-E9) + (P10-E10) + (P11-E11) + (P12-E12)]/4
Economic	=[(P13-E13) + (P14-E14) + (P15-E15) + (P16-E16)]/4
Empathy	=[(P17-E17) + (P18-E18) + (P19-E19) + (P20-E20)]/4
Tangibles	=[(P21-E21) + (P22-E22) + (P23-E23) + (P24-E24)]/4
Overall	=[(P1-E1)+...+(P24-E24)]/24

### 2.6 Statistical analysis

Statistical analyses were performed using SPSS 26.0 (IBM Corp., Armonk, NY, USA). Continuous variables were summarized as mean ± standard deviation (SD), and categorical variables as frequencies and percentages. Mean ± SD SQ scores were calculated by survey year and demographic subgroup to describe the overall level and distribution of smart healthcare service quality. In univariable analyses, independent-samples *t*-tests and analysis of variance (ANOVA) were used to compare SQ scores across demographic groups. Variables with *P* < 0.05 in the univariable analyses were entered into the multivariable linear regression model.

In multivariable linear regression analyses, SQ score was specified as the dependent variable, whereas survey year, age, education, occupation, monthly income, residence, and hospital were entered as independent variables. The hospital was included as a fixed effect to account for stable, hospital-specific characteristics, such as smart-healthcare infrastructure, hospital scale, management, and local socioeconomic context, that could otherwise confound estimates of change over time. This approach controls for such differences across hospitals, so that the survey-year coefficient reflects within-hospital change rather than shifts in hospital composition (18). The regression coefficient for the survey year reflects changes in SQ scores over time after controlling for demographic characteristics and hospital. Multicollinearity was assessed before model fitting using variance inflation factors (VIFs). VIFs ranged from 1.030 to 2.188, indicating no evidence of problematic multicollinearity.

To examine whether there were differences in the temporal changes in SQ scores among patients with different demographic characteristics. Separate interaction models were fitted by adding an interaction term between survey year and each demographic characteristic to the adjusted regression model. Adjusted marginal means were estimated for each subgroup, and subgroup-specific temporal patterns were visualized using predicted marginal mean plots. All tests were two-sided, and *P* < 0.05 was considered statistically significant.

## 3 Results

### 3.1 Participant characteristics and univariable analyses

A total of 900 patients were surveyed across the three waves. Valid questionnaires were obtained from 282, 286, and 287 participants in 2019, 2021, and 2025, respectively, corresponding to valid response rates of 94.0%, 95.3%, and 95.7%. Overall, 855 valid questionnaires were obtained (valid response rate = 95%). The remaining 45 questionnaires were excluded due to incomplete responses. The distribution by year was 18 in 2019, 14 in 2021, and 13 in 2025. Among them, 414 were male (48.4%), and 441 were female (51.6%). Most participants were younger than 60 years (74.3%), whereas 25.7% were aged 60 years or older. 48.2% of patients had a high school or vocational college education, 48% were employed, and 26.4% had a personal monthly income of 3,500–5,000 CNY. Urban employee medical insurance (42.5%) and urban residents' healthcare insurance (49.4%) were the primary insurance schemes among the surveyed patients. Most participants were local residents (90.8%). The mean SQ score improved from −0.72 ± 0.60 in 2019 to −0.46 ± 0.47 in 2025. Univariable analyses showed that SQ scores differed significantly by age, educational attainment, occupation, monthly income, place of residence, and survey year (*P* < 0.05), whereas no significant differences were observed by gender and insurance type (*P* > 0.05). Please refer to Table 2 for detailed results. Participant characteristics by survey year are presented in Supplementary Table S1.

**Table 2:** Participant characteristics and univariable analyses.

Variables	Category	*n* (%)	Mean SQ score (SD)	*t*/*F*	*P*-value
Gender	Male	414 (48.4)	−0.56 ± 0.54	1.081	0.28
	Female	441 (51.6)	−0.61 ± 0.56		
Age	< 60 years	635 (74.3)	−0.51 ± 0.55	7.031	< 0.001
	≥60 years	220 (25.7)	−0.79 ± 0.49		
Education	Middle school or below	208 (24.3)	−0.50 ± 0.60	6.342	0.002
	High school/College	412 (48.2)	−0.58 ± 0.53		
	Bachelor's degree or above	235 (27.5)	−0.68 ± 0.53		
Occupation	Employed	410 (48.0)	−0.54 ± 0.54	10.937	< 0.001
	Retired	143 (16.7)	−0.78 ± 0.55		
	Unemployed/Other	302 (35.3)	−0.56 ± 0.55		
Monthly income	No income	224 (26.2)	−0.70 ± 0.60	5.403	0.001
	< 3,500 CNY	201 (23.5)	−0.55 ± 0.51		
	3,500–5,000 CNY	226 (26.4)	−0.59 ± 0.57		
	>5,000 CNY	204 (23.9)	−0.50 ± 0.51		
Insurance	Self-financed	50 (5.8)	−0.56 ± 0.54	0.61	0.609
	Urban employee healthcare insurance	363 (42.5)	−0.56 ± 0.53		
	Urban residents' healthcare insurance	422 (49.4)	−0.61 ± 0.58		
	Business Insurance	20 (2.3)	−0.52 ± 0.48		
Residence	Local	776 (90.8)	−0.60 ± 0.55	−2.469	0.014
	Nonlocal	79 (9.2)	−0.44 ± 0.50		
Survey year	2019	282 (33.0)	−0.72 ± 0.60	16.439	< 0.001
	2021	286 (33.5)	−0.57 ± 0.54		
	2025	287 (33.6)	−0.46 ± 0.47		

### 3.2 SQ Scores for the 24 smart healthcare service quality items

The mean score for smart healthcare service quality across the three survey waves improved over time, increasing from −0.72 ± 0.60 in 2019 to −0.57 ± 0.54 in 2021 and −0.46 ± 0.47 in 2025; differences among survey waves were statistically significant (*P* < 0.001). SQ scores were negative at all three survey waves, indicating that perceived service quality remained below patients' expectations, although the gap narrowed over time.

The results of the analysis of variance for the 24 items showed that, with the exception of the four items in the economic dimension (items 13–16, *P* > 0.05), the remaining 20 items showed statistically significant differences in SQ scores across survey waves (*P* < 0.05), indicating that patients' perceived quality of most smart healthcare service items differed significantly across survey waves. Please refer to Table 3 for detailed results.

**Table 3:** SQ scores for the 24 items assessing smart healthcare service quality.

Dimensions	Items	2019 (mean±SD)	2021 (mean±SD)	2025 (mean±SD)	*F*	*P-*value
Reliability	1. Ease of use of smart devices	−0.61 ± 0.77	−0.57 ± 0.94	−0.37 ± 0.76	6.494	0.002
	2. Accuracy of platform information	−0.59 ± 0.69	−0.52 ± 0.85	−0.37 ± 0.79	5.891	0.003
	3. Online feedback and complaint channels	−1.06 ± 0.80	−0.61 ± 0.98	−0.47 ± 0.88	34.354	< 0.001
	4. Fee information transparency	−0.68 ± 0.72	−0.51 ± 0.92	−0.43 ± 0.80	6.572	0.001
	Subtotal	−0.73 ± 0.52	−0.54 ± 0.71	−0.41 ± 0.61	19.739	< 0.001
Assurance	5. Efficient intelligent diagnosis	−0.82 ± 0.75	−0.52 ± 0.87	−0.37 ± 0.77	23.769	< 0.001
	6. Online medical services	−0.90 ± 0.80	−0.55 ± 0.98	−0.54 ± 0.89	14.558	< 0.001
	7. Payment platform security	−0.61 ± 0.77	−0.41 ± 0.85	−0.29 ± 0.73	11.963	< 0.001
	8. Timely test reminders	−0.70 ± 0.75	−0.42 ± 0.89	−0.36 ± 0.69	14.954	< 0.001
	Subtotal	−0.76 ± 0.59	−0.47 ± 0.65	−0.39 ± 0.54	29.846	< 0.001
Responsiveness	9. Reduced waiting time	−0.78 ± 0.95	−0.51 ± 0.81	−0.42 ± 0.80	13.464	< 0.001
	10. Online doctor communication	−0.72 ± 0.98	−0.46 ± 0.94	−0.48 ± 0.91	6.370	0.002
	11. Healthcare problem resolution	−0.54 ± 0.85	−0.43 ± 0.91	−0.32 ± 0.78	4.650	0.010
	12. Test result access	−0.48 ± 1.03	−0.58 ± 0.96	−0.37 ± 0.72	3.910	0.020
	Subtotal	−0.63 ± 0.77	−0.47 ± 0.68	−0.40 ± 0.64	7.889	< 0.001
Economic	13. Reasonable drug costs	−0.92 ± 1.11	−0.77 ± 1.03	−0.85 ± 0.90	1.454	0.234
	14. Reasonable cost of inspection	−0.98 ± 1.04	−0.83 ± 1.04	−0.86 ± 0.89	1.780	0.169
	15. Reduction of additional costs	−0.90 ± 0.98	−0.83 ± 1.00	−0.83 ± 0.87	0.509	0.601
	16. Reasonable cost of accommodation and food	−0.91 ± 1.10	−0.81 ± 0.99	−0.74 ± 0.92	1.911	0.149
	Subtotal	−0.93 ± 1.01	−0.81 ± 0.91	−0.82 ± 0.80	1.39	0.250
Empathy	17. Privacy protection	−0.67 ± 0.90	−0.58 ± 0.87	−0.39 ± 0.72	7.976	< 0.001
	18. Informed consent and data communication	−0.51 ± 1.03	−0.63 ± 0.94	−0.43 ± 0.72	3.491	0.031
	19. Optimization of healthcare processes	−0.63 ± 1.00	−0.55 ± 0.93	−0.41 ± 0.77	4.424	0.012
	20. Personalized service provision	−0.80 ± 0.93	−0.60 ± 0.86	−0.44 ± 0.76	12.573	< 0.001
	Subtotal	−0.65 ± 0.79	−0.59 ± 0.73	−0.42 ± 0.57	8.65	< 0.001
Tangibles	21. Adequate smart devices	−0.72 ± 1.00	−0.46 ± 0.87	−0.30 ± 0.75	16.672	< 0.001
	22. Department information transparency	−0.66 ± 0.88	−0.60 ± 0.79	−0.40 ± 0.80	7.962	< 0.001
	23. Appropriateness of equipment layout	−0.52 ± 1.06	−0.53 ± 0.94	−0.33 ± 0.75	4.381	0.013
	24. Accessible inpatient records	−0.65 ± 1.07	−0.59 ± 0.86	−0.35 ± 0.72	8.658	< 0.001
	Subtotal	−0.64 ± 0.81	−0.55 ± 0.71	−0.34 ± 0.60	12.787	< 0.001
Overall		−0.72 ± 0.60	−0.57 ± 0.54	−0.46 ± 0.47	16.439	< 0.001

### 3.3 Temporal trends in the six dimensions of smart healthcare service quality

As shown in Figure 1 and Table 3, SQ scores improved to varying degrees across five dimensions with the exception of economic. The largest improvements were observed in assurance (from −0.76 to −0.39, *P* < 0.001), reliability (from −0.73 to −0.41, *P* < 0.001), and tangibles (from −0.64 to −0.34, *P* < 0.001). Responsiveness (from −0.63 to −0.40, *P* < 0.001) and empathy (from −0.65 to −0.42, *P* < 0.001) also showed significant improvement. The score for the economic dimension showed the least variation across the 3 years (−0.93 in 2019, −0.81 in 2021, and −0.82 in 2025, *P* > 0.05) and remained the lowest among all dimensions, indicating that patients' perceived quality of services related to medical costs has improved only marginally and therefore remains a priority for improving smart healthcare service quality. Mean expectation and perception scores underlying these gaps for each dimension and survey wave are reported in Supplementary Table S2.

**Figure 1:**
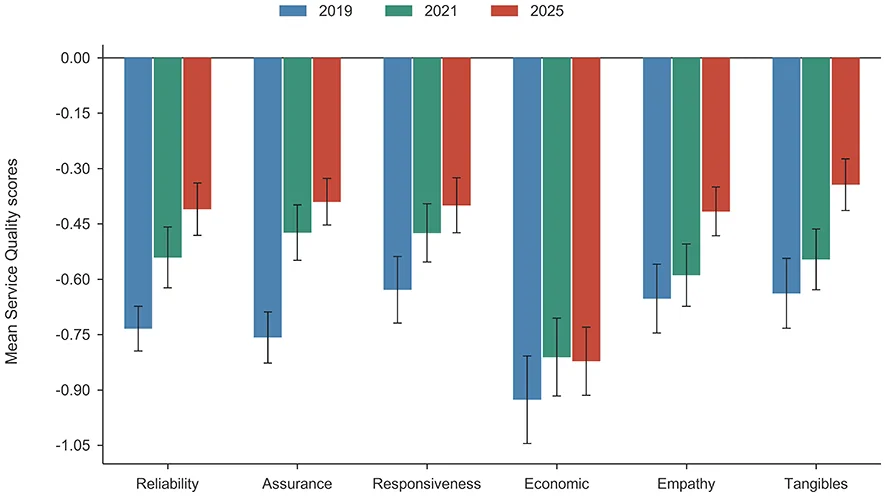
Trends in SQ scores across six dimensions of smart healthcare service quality, 2019–2025. Error bars represent 95% confidence intervals.

### 3.4 Analysis of differences in patients' evaluations of smart healthcare service quality

Using the score for smart healthcare service quality as the dependent variable, a multivariable linear regression model was fitted with survey year, age, education, occupation, monthly income, residence, and hospital as categorical fixed factors. The results (Table 4) show that, after controlling for other covariates, patients' age, education, monthly income, residence, and survey year were independently associated with SQ score (*P* < 0.05). Compared with 2019, adjusted SQ scores were 0.122 points higher in 2021 (*B* = 0.122, 95% CI: 0.035 to 0.209, *P* = 0.006) and 0.234 points higher in 2025 (*B* = 0.234, 95% CI: 0.148 to 0.319, *P* < 0.001), indicating an overall improvement in perceived smart healthcare service quality across the three survey waves after adjustment.

**Table 4:** Multiple linear regression showing differences in mean SQ scores across different predictor variables, adjusted for hospital fixed effects.

Variables	*B*	95% CI	*P-*value
Age (Reference:<60 years)			
≥60 years	−0.212	−0.306, −0.117	< 0.001
Education (Reference: middle school or below)			
High school/College	0.064	−0.033, 0.161	0.197
Bachelor's degree or above	−0.117	−0.214, −0.019	0.019
Occupation (Reference: employed)			
Retired	−0.057	−0.176, 0.062	0.346
Unemployed/Other	0.036	−0.056, 0.129	0.442
Monthly income (Reference: no income)			
< 3,500 CNY	0.122	0.019, 0.226	0.021
3,500–5,000 CNY	0.090	−0.018, 0.197	0.102
>5,000 CNY	0.160	0.040, 0.279	0.009
Residence (Reference: local)			
Nonlocal	0.137	0.016, 0.258	0.026
Survey year (Reference: 2019)			
2021	0.122	0.035, 0.209	0.006
2025	0.234	0.148, 0.319	< 0.001

Hospital fixed effects were included (Hospital 1 as the reference) to account for between-hospital differences. Overall model: *P* < 0.001; *R*^2^ = 0.147.

Regarding demographic factors, compared with patients under 60 years of age, patients aged 60 years or older had SQ scores that were 0.212 points lower (95% CI: −0.306 to −0.117). Compared with patients with a junior high school education or lower, participants with a bachelor's degree or higher had lower SQ scores (*B* = −0.117, 95% CI: −0.214 to −0.019). In terms of monthly income, compared with patients with no income, those with a monthly income of < 3,500 CNY (*B* = 0.122, 95% CI: 0.019 to 0.226) and those with a monthly income of >5,000 CNY (*B* = 0.160, 95% CI: 0.040 to 0.279) had higher SQ scores. Nonlocal patients had higher SQ scores than local participants (*B* = 0.137, 95% CI: 0.016 to 0.258). Occupation was not independently associated with SQ score after covariate adjustment (*P* > 0.05).

### 3.5 Temporal changes in adjusted smart healthcare service quality scores across demographic subgroups

After adjusting for demographic covariates, the interaction effects between survey year and age (*F* = 11.345, *P* < 0.001), education (*F* = 15.857, *P* < 0.001), occupation (*F* = 3.500, *P* = 0.008), and monthly income (*F* = 3.303, *P* = 0.003) were all statistically significant, indicating that the temporal changes in SQ scores over time differed significantly among these subgroups (Figure 2). The interaction effect between survey year and place of residence was not statistically significant (*F* = 2.871, *P* = 0.057). Detailed interaction results and adjusted changes are presented in Supplementary Table S3. The adjusted marginal means are provided in Supplementary Table S4.

**Figure 2:**
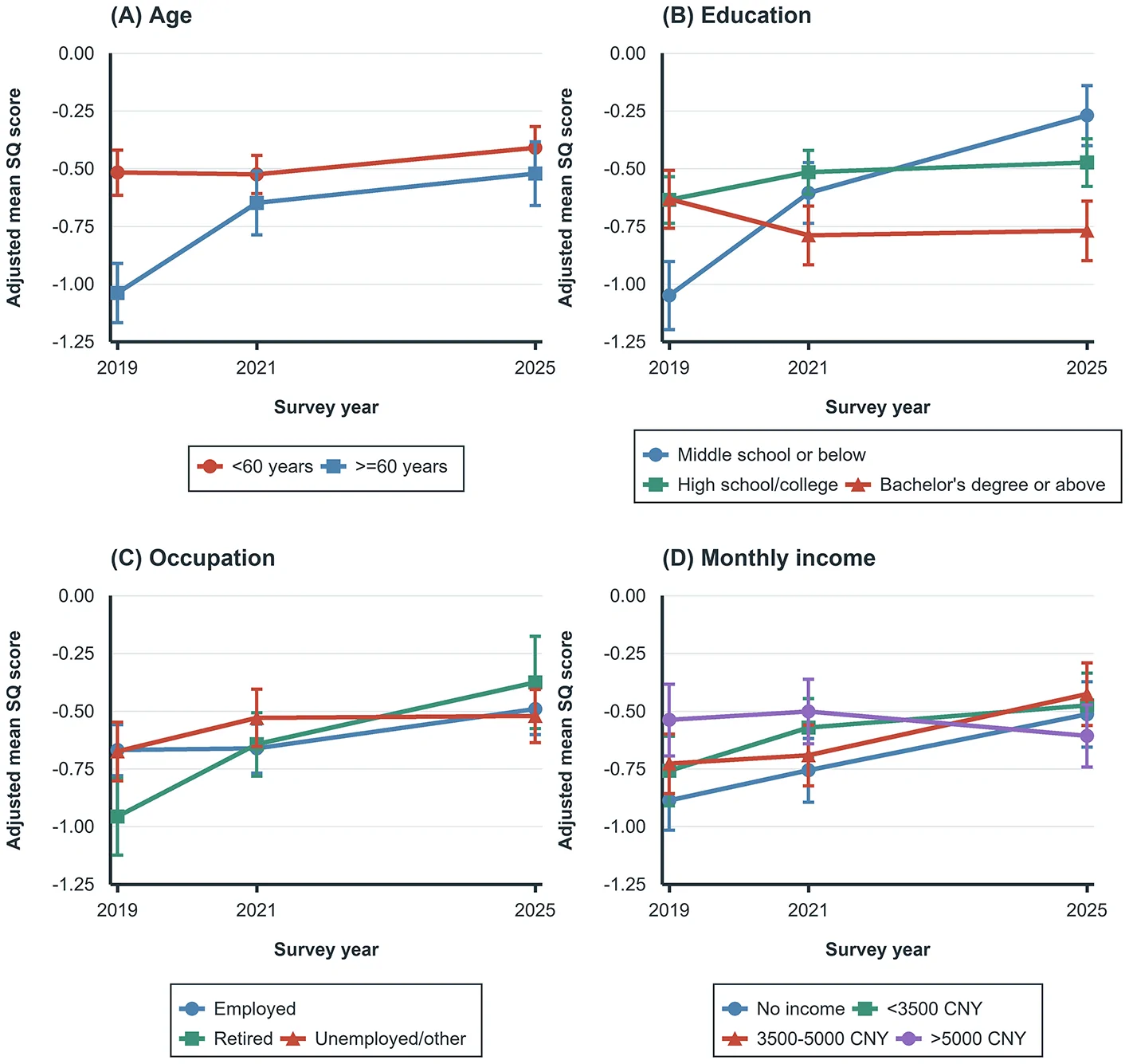
Adjusted mean smart healthcare service quality scores with 95% confidence intervals by age, education, occupation, and monthly income, 2019–2025.

Age: scores improved modestly among participants younger than 60 years, whereas the estimated improvement was larger among those aged 60 years or older. The between-group difference narrowed numerically over time.

Education: patients with a junior high school education or lower showed the most pronounced improvement, whereas scores for patients with a bachelor's degree or higher stabilized after a decline in 2021, exhibiting a different trajectory from other educational groups.

Occupation: retired patients had the lowest scores in 2019 but showed the greatest improvement by 2025; the score trajectories of employed and unemployed/other patients were relatively similar.

Monthly income: participants with no income showed a substantial improvement, while those with a monthly income of >5,000 CNY experienced a slight, non-significant decline in scores in 2025.

## 4 Discussion

### 4.1 Gradual improvement in the quality of smart healthcare services at county-level public hospitals in Chongqing

This study found that inpatients' perceived smart healthcare service quality scores at county-level public hospitals in Chongqing improved from 2019 to 2025. The overall SQ score increased from −0.72 to −0.46, and this improvement remained statistically significant after adjustment for demographic characteristics. This improvement may be related to ongoing national and local efforts to promote high-quality public-hospital development and smart healthcare initiatives. In particular, Chongqing has gradually implemented its smart healthcare action plan in recent years and vigorously promoted the establishment of closely integrated county-level healthcare communities, supporting systematic investment in smart equipment, information platforms, and diagnostic and treatment process optimization. These investments have strengthened hospitals' digital infrastructure and streamlined patient pathways from appointment scheduling and admission to testing and payment. Improvements in hospital digitalization enabled patients to access more convenient and efficient services throughout the care journey, thereby improving perceived service quality.

It is worth noting that although overall SQ scores improved, SQ gap scores remained negative across all six dimensions and 24 items. This finding is consistent with previous studies conducted in China (16, 20) and other countries (21, 22). This suggests that smart healthcare services at county-level public hospitals in Chongqing have not yet fully met patients' actual needs, with some remaining weaknesses and considerable room for improvement. As patients become more familiar with smart healthcare services and accumulate experience using them, their expectations may also increase. Investment in technical infrastructure alone is unlikely to fully close the gap between patients' expectations and perceptions. In addition to continued investment in digital infrastructure, patient-centered service-process design and improvements in the usability and comfort of smart healthcare services may help narrow this gap.

### 4.2 Dimension changes in smart healthcare service quality

The reliability dimension covers the ease of use of smart devices, the accuracy of platform information, online channels for reviews and complaints, and the transparency of fee information. The score for this dimension showed a substantial improvement. This improvement may reflect efforts to improve device usability, enhance information transparency, and maintain accessible feedback and complaint channels. Patient's evaluation of the platform's fundamental credibility has been continuously improving (23).

The assurance dimension covers the efficiency of smart diagnosis and treatment, the accessibility of online medical services, payment security, and the timeliness of test reminders. This dimension showed the most significant improvement among the six dimensions, which may be related sustained investment by county-level public hospitals in Chongqing in improving smart diagnostic and treatment efficiency and expanding online service availability. Improvements in hospitals' technical capabilities have had a positive impact on patient perceptions (24). The continuous improvement of information systems and online services has also strengthened patients' confidence in the quality and safety of medical care (25). Notably, the accessibility of online medical services (item 6) showed the largest numerical increase between 2019 and 2021 (from −0.90 to −0.55), which may reflect expanded use of internet-based care during the COVID-19 pandemic and greater patient acceptance of online care models (26, 27).

The responsiveness dimension encompasses reduced waiting times, online doctor-patient communication, resolution of medical issues, and access to test results. The score for this dimension showed a steady upward trend. The wider availability of appointment scheduling systems and mobile report notifications may have reduced waiting times and improved access to information, thereby contributing to improvement in this dimension (28). However, improvements in the area of online clinician-patient communication have been relatively limited. Future initiatives could prioritize in-hospital instant messaging, intelligent consultation systems, and bedside interactive terminals to improve the quality and efficiency of clinician-patient communication (29).

The empathy dimension covers the protection of private information, informed consent for data collection, personalized optimization of the healthcare process, and the provision of personalized services. Improvements in privacy protection and informed consent for data collection reflect an increased level of standardization in data security management, which is related to China's continuous strengthening of legislation and regulatory oversight regarding healthcare data security in recent years (30). However, the score for personalized services remains low. Accurately identifying diverse patient needs (31) and delivering genuinely personalized services within standardized information systems remain substantial challenges, representing a key direction for the future development of smart healthcare services (32).

The tangibles dimension covers the adequacy of smart devices, transparency of departmental information, rationality of equipment layout, and the accessibility of inpatient records. The magnitude of improvement in this dimension was second only to that of the assurance dimension. This finding indicates that in recent years, county-level public hospitals have achieved certain results in terms of investment in intelligent hardware facilities. The widespread adoption of self-service terminals, mobile applications, and electronic inpatient records may have improved patients' perceptions of hospital digital infrastructure (33).

The economic dimension encompasses four items: the reasonableness of medication costs, the reasonableness of diagnostic test costs, the reduction of unnecessary charges, and the reasonableness of room and board costs. Compared with the other dimensions, the economic dimension changed the least and was the only dimension without statistically significant improvement, a finding consistent with the conclusions of similar studies in China (16). In theory, hospitals could leverage intelligent prescription review systems to standardize medication use and promote reasonable drug costs, and use regional smart-platform sharing to achieve mutual recognition of examination results, thereby avoiding redundant testing (19). Online follow-up consultations through internet hospitals could similarly help reduce the additional travel and accommodation costs incurred by patients traveling to and from the hospital after discharge. However, the survey showed that patients rated the reasonableness of these cost-related items relatively low. This may be attributable to the fact that, in the informatization development of county-level medical consortiums, data-interface barriers and differences in quality-control standards have not yet been fully eliminated, preventing mutual recognition of examination results, while weaknesses in foundational information systems and limited interconnectivity among regional information platforms (34). In addition, patients at county-level public hospitals in Chongqing generally have lower income and education, with comparatively inelastic healthcare needs (35), most continue to prefer traditional in-person care, meaning that indirect costs such as travel and accommodation were not substantially reduced. Together, these factors may contribute to patients' negative evaluations of the economic dimension.

### 4.3 Differences in perceived smart healthcare service quality across patient subgroups

Multivariable linear regression and interaction analyses indicated statistically significant interactions between survey year and age, education, occupation, and monthly income, indicating that patient groups with different characteristics exhibit significant differences in their evaluations of smart healthcare service quality and in the trajectories of these evaluations over time.

Regarding age, in 2019, participants aged 60 years or older reported lower SQ scores than those younger than 60 years; however, their scores improved more markedly across subsequent survey waves, and by 2025, the gap between the two groups had narrowed markedly. The initially lower ratings among older patients may be related to the greater technical barriers they face when using smart devices and digital service platforms. Previous studies suggest that declines in visual, auditory, and cognitive function may make smart healthcare services more difficult for older adults to use (36). The gradual narrowing of the gap between the two groups may be related to the advancement of age-friendly adaptations to smart healthcare services; however, older patients' ratings remain lower than those of younger patients, indicating that the digital divide persists. Efforts to enhance age-friendly smart healthcare services should be continuously deepened, including simplifying operational procedures, assigning in-person support staff, and developing family account functions (37).

In terms of education, participants with a bachelor's degree or higher showed a distinct trajectory: their adjusted SQ scores declined in 2021 and remained comparatively low in 2025. In contrast, participants with a middle school education or below showed the largest improvement over time. This may be because, in the initial phase of smart healthcare implementation, this group rated the new technology favorably owing to the novelty it offered, whereas the digital improvements introduced subsequently provided relatively limited additional benefit for this group. In contrast, patients with lower educational attainment had lower baseline scores, so the same improvements provided them with greater room for improvement. In addition, patients with higher educational attainment possess greater health literacy and digital literacy and have more exposure to more mature digital services in other contexts. Consequently, their expectations for smart healthcare service may be higher, making it more difficult to narrow the gap between perception and expectation (14). This suggests that while hospitals enhance technical infrastructure and service platform functionality, they must simultaneously address the differentiated needs of patients with higher educational attainment in areas such as service personalization, information transparency, and data security.

In terms of occupation, retired participants had the lowest SQ scores in 2019 but showed the largest improvement by 2025, were similar to the scores of employed participants. The initially low ratings among retired patients are consistent with their older age profile and the challenges they face in adapting to technology; their significant improvement aligns with the trend observed among older patients in the age subgroup. This pattern is consistent with the possibility that age-friendly adaptations may have contributed to improved scores among retired participants.

In terms of monthly income, patients with no income had the lowest ratings in 2019 and showed the greatest improvement, while patients with a monthly income of >5,000 CNY experienced a slight decline by 2025. After adjusting for demographic covariates, this decline among patients with a monthly income of >5,000 CNY was not statistically significant (adjusted change, 2019–2025: −0.069, 95% CI: −0.245 to 0.108, *P* = 0.445). The marked improvement among low-income patients may be related to their lower baseline ratings and greater room for improvement. Broad service improvements, such as streamlined inpatient processes, may be more readily perceived by this group. The numerical decline among high-income patients, by contrast, may reflect that this group already had comparatively higher baseline scores than other income groups, leaving less room for further measurable improvement, and that smart healthcare services at county-level public hospitals have not yet fully met the needs of this group.

In addition, although the interaction between place of residence and survey year was not statistically significant, nonlocal patients had significantly higher SQ scores than local patients in the multivariable model. One possible explanation is a positive selection effect: unlike local patients, who typically receive care at the nearest hospital by default, nonlocal patients generally must actively weigh multiple considerations before choosing to seek care outside their home area, and this decision process itself may reflect greater initial trust and more positive expectations regarding the care they will receive (38). Moreover, nonlocal patients may lack the accompaniment and assistance of family members, and may therefore rely more heavily on self-service terminals, online payment, and information inquiry functions to independently navigate the care process, potentially making them more directly aware of the convenience these smart healthcare services provide. This may represent an additional contributing factor.

### 4.4 Limitations

The sample was drawn from only six county-level public hospitals in Chongqing, which may limit the generalizability of the findings; future studies should include a broader range of county-level public hospitals to improve representativeness. In addition, the three survey waves did not involve the same cohort of patients but instead employed a repeated cross-sectional design; individual-level changes in perceived service quality could therefore not be assessed, and causal inferences cannot be drawn from the observed associations. Furthermore, although hospital fixed effects were used to control for stable differences among the six participating hospitals and the core services assessed remained relatively consistent across waves, the study did not collect detailed hospital-level measures of resources, digital-service changes in implementation intensity over time. Finally, the regression model explained a relatively modest proportion of variance in SQ scores (*R*^2^ = 0.147), which is not uncommon in patient experience research; other unmeasured factors, such as disease severity, length of hospital stay, and patients' prior healthcare experiences, may further explain variation in perceived service quality, and patients' digital health literacy and individual out-of-pocket cost burden may also be relevant but unmeasured factors. Future studies should consider incorporating such clinical, experiential, and cost-related variables to improve model explanatory power.

## 5 Conclusion

Based on the results of three repeated cross-sectional survey waves, the scores for the perceived quality of smart healthcare services among inpatients at county-level public hospitals in Chongqing from 2019 to 2025 showed an upward trend across the three survey waves, suggesting that the development of smart healthcare may has improved the patient experience. However, patients' evaluations of cost-related services remained relatively low throughout the study period, with only limited improvement, indicating that patients' concerns about the reasonableness of medical costs may be a key factor limiting the improvement in the quality of smart healthcare services.

There were differences in the smart healthcare service quality scores and their temporal patterns among patients with different demographic characteristics. Patients aged 60 years or older and those with a bachelor's degree or higher gave relatively lower overall ratings; patients with no income gave lower ratings in the early surveys, but their scores improved significantly. Service quality scores across different income groups exhibited heterogeneous changes over time; notably, the high-income group experienced a slight decline in 2025. These findings suggest that addressing the differentiated needs of various patient groups, promoting mutual recognition of diagnostic test results across institutions, refining cost-control mechanisms, and strengthening age-friendly service design and information system integration may represent promising directions for improving the quality of smart healthcare services and the patient experience at county-level public hospitals.

Future research could expand the sample coverage to include more county-level public hospitals and, through qualitative interviews, explore in depth the specific reasons for differences in the evaluation of smart healthcare service quality and its trajectory across different populations, thereby providing a basis for targeted, stratified, and categorized interventions.

## Data Availability

The original contributions presented in the study are included in the article, further inquiries can be directed to the corresponding author.
