# Supplementary material for: Changes in inpatients' perceptions of the quality of smart healthcare services at county-level public hospitals in Chongqing: a repeated cross-sectional study, 2019-2025
Source: Front Public Health. 2026 Oct 21;14:1942631. doi: 10.3389/fpubh.2026.1942631 (PMC13731293; doi:10.3389/fpubh.2026.1942631)
Supplement: Supplementary file 1 [file Table_1.docx]

Supplementary Material

Supplementary Table S1. Participant characteristics by survey year

| Variables | Category | 2019  (n=282) | Percentage  (%) | 2021  (n=286) | Percentage  (%) | 2025  (n=287) | Percentage  (%) |
| --- | --- | --- | --- | --- | --- | --- | --- |
| Gender | Male | 142 | 50.4 | 128 | 44.8 | 144 | 50.2 |
|  | Female | 140 | 49.6 | 158 | 55.2 | 143 | 49.8 |
| Age | <60 years | 193 | 68.4 | 225 | 78.7 | 217 | 75.6 |
|  | ≥60 years | 89 | 31.6 | 61 | 21.3 | 70 | 24.4 |
| Education | Middle school or below | 56 | 19.9 | 71 | 24.8 | 81 | 28.2 |
|  | High school/College | 146 | 51.8 | 138 | 48.3 | 128 | 44.6 |
|  | Bachelor’s degree or above | 80 | 28.4 | 77 | 26.9 | 78 | 27.2 |
| Occupation | Employed | 135 | 47.9 | 134 | 46.9 | 141 | 49.1 |
|  | Retired | 48 | 17 | 65 | 22.7 | 30 | 10.5 |
|  | Unemployed/Other | 99 | 35.1 | 87 | 30.4 | 116 | 40.4 |
| Monthly income | No income | 88 | 31.2 | 65 | 22.7 | 71 | 24.7 |
|  | <3500 CNY | 57 | 20.2 | 80 | 28 | 64 | 22.3 |
|  | 3500-5000 CNY | 82 | 29.1 | 75 | 26.2 | 69 | 24 |
|  | >5000 CNY | 55 | 19.5 | 66 | 23.1 | 83 | 28.9 |
| Insurance | Self-financed | 23 | 8.2 | 16 | 5.6 | 11 | 3.8 |
|  | Urban employee healthcare insurance | 126 | 44.7 | 119 | 41.6 | 118 | 41.1 |
|  | Urban residents'healthcare insurance | 131 | 46.5 | 141 | 49.3 | 150 | 52.3 |
|  | Business Insurance | 2 | 0.7 | 10 | 3.5 | 8 | 2.8 |
| Residence | Local | 267 | 94.7 | 245 | 85.7 | 264 | 92 |
|  | Nonlocal | 15 | 5.3 | 41 | 14.3 | 23 | 8 |

Supplementary Table S2. Mean expectation and perception scores by dimension and survey wave

| Dimensions | 2019(mean±SD) | | 2021(mean±SD) | | 2025(mean±SD) | |
| --- | --- | --- | --- | --- | --- | --- |
|  | Expectation(E) | Perception (P) | Expectation(E) | Perception (P) | Expectation(E) | Perception (P) |
| Reliability | 4.62±0.41 | 3.89±0.45 | 4.47±0.58 | 3.93±0.70 | 4.38±0.57 | 3.96±0.64 |
| Assurance | 4.69±0.40 | 3.93±0.51 | 4.46±0.53 | 3.99±0.68 | 4.51±0.50 | 4.12±0.58 |
| Responsiveness | 4.51±0.46 | 3.88±0.61 | 4.48±0.55 | 4.01±0.75 | 4.51±0.52 | 4.11±0.68 |
| Economic | 4.44±0.66 | 3.52±0.70 | 4.62±0.53 | 3.81±0.90 | 4.41±0.65 | 3.60±0.69 |
| Empathy | 4.46±0.50 | 3.81±0.58 | 4.55±0.53 | 3.96±0.82 | 4.50±0.53 | 4.08±0.66 |
| Tangibles | 4.45±0.50 | 3.81±0.62 | 4.53±0.53 | 3.98±0.78 | 4.53±0.56 | 4.15±0.71 |
| Overall | 4.53±0.34 | 3.81±0.50 | 4.52±0.44 | 3.95±0.66 | 4.47±0.44 | 4.01±0.55 |

Supplementary Table S3. Interaction tests and adjusted changes in service quality scores.

| Interaction | F | *P*-value for interaction | Subgroup | Adjusted change, 2019-2025 (95% CI) | *P*-value for change |
| --- | --- | --- | --- | --- | --- |
| Survey year x Age | 11.345 | <0.001 | <60 years | 0.107 (0.006, 0.208) | 0.037 |
|  |  |  | ≥60 years | 0.517 (0.354, 0.680) | <0.001 |
| Survey year x Education | 15.857 | <0.001 | Middle school or below | 0.780 (0.607, 0.952) | <0.001 |
|  |  |  | High school/college | 0.162 (0.041, 0.283) | 0.009 |
|  |  |  | Bachelor's degree or above | -0.137 (-0.295, 0.021) | 0.089 |
| Survey year x Occupation | 3.500 | 0.008 | Employed | 0.178 (0.054, 0.302) | 0.005 |
|  |  |  | Retired | 0.582 (0.345, 0.818) | <0.001 |
|  |  |  | Unemployed/other | 0.154 (0.014, 0.294) | 0.032 |
| Survey year x  Monthly income | 3.303 | 0.003 | No income | 0.373 (0.211, 0.535) | <0.001 |
|  |  |  | <3500 CNY | 0.284 (0.098, 0.470) | 0.003 |
|  |  |  | 3500-5000 CNY | 0.301 (0.135, 0.467) | <0.001 |
|  |  |  | >5000 CNY | -0.069 (-0.245, 0.108) | 0.445 |
| Survey year x Residence | 2.871 | 0.057 |  |  |  |

Note: Five separate multivariable general linear models were fitted, each containing all demographic variables as fixed factors and one survey year-by-subgroup interaction. Differences are calculated as 2025 minus 2019 estimated marginal means.

Supplementary Table S4. Adjusted marginal mean service quality scores by survey year and demographic subgroup.

| Subgroup variable | Category | 2019 adjusted  mean (95% CI) | 2021 adjusted  mean (95% CI) | 2025 adjusted  mean (95% CI) |
| --- | --- | --- | --- | --- |
| Age | <60 years | -0.517 (-0.615, -0.419) | -0.525 (-0.608, -0.442) | -0.410 (-0.502, -0.318) |
|  | ≥60 years | -1.039 (-1.167, -0.910) | -0.649 (-0.787, -0.510) | -0.522 (-0.660, -0.384) |
| Education | Middle school or below | -1.049 (-1.198, -0.901) | -0.605 (-0.736, -0.473) | -0.270 (-0.400, -0.140) |
|  | High school/college | -0.635 (-0.736, -0.535) | -0.516 (-0.611, -0.421) | -0.474 (-0.576, -0.371) |
|  | Bachelor's degree or above | -0.632 (-0.757, -0.507) | -0.789 (-0.916, -0.661) | -0.769 (-0.898, -0.640) |
| Occupation | Employed | -0.669 (-0.779, -0.559) | -0.662 (-0.768, -0.555) | -0.491 (-0.601, -0.380) |
|  | Retired | -0.957 (-1.124, -0.790) | -0.644 (-0.781, -0.506) | -0.375 (-0.575, -0.175) |
|  | Unemployed/other | -0.675 (-0.802, -0.547) | -0.529 (-0.653, -0.405) | -0.521 (-0.636, -0.406) |
| Monthly income | No income | -0.887 (-1.016, -0.758) | -0.756 (-0.894, -0.619) | -0.514 (-0.655, -0.373) |
|  | <3500 CNY | -0.759 (-0.909, -0.609) | -0.571 (-0.698, -0.445) | -0.475 (-0.615, -0.335) |
|  | 3500-5000 CNY | -0.728 (-0.857, -0.598) | -0.691 (-0.824, -0.559) | -0.426 (-0.562, -0.291) |
|  | >5000 CNY | -0.538 (-0.693, -0.383) | -0.501 (-0.641, -0.361) | -0.607 (-0.742, -0.472) |

Note: Each model included survey year, age, education, occupation, monthly income, and residence as fixed factors, together with the relevant survey year-by-subgroup interaction. EMMs were averaged equally over the levels of the remaining fixed factors.
